# Hydrogen Production from Formic Acid Decomposition Promoted by Gold Nanoparticles Supported on a Porous Polymer Matrix

**DOI:** 10.1021/acs.energyfuels.5c01537

**Published:** 2025-07-10

**Authors:** Matteo Diglio, Irene Contento, Salvatore Impemba, Enrico Berretti, Paolo Della Sala, Giuseppina Oliva, Vincenzo Naddeo, Stefano Caporali, Ana Primo, Carmen Talotta, Carmine Gaeta, Carmine Capacchione, Alfonso Grassi, Antonio Buonerba

**Affiliations:** † Department of Chemistry and Biology “Adolfo Zambelli”, 19028 University of Salerno, Via Giovanni Paolo II, 132, 84084 Fisciano (SA), Italy; ‡ Interuniversity Consortium of Chemical Reactivity and Catalysis (CIRCC), Via Celso Ulpiani, 27, 70126 Bari, Italy; § NANO_MATES (Research Centre for Nanomaterials and Nanotechnology, Via Giovanni Paolo II, 132, 84084 Fisciano (SA), Italy; ∥ Institute of Chemistry of the Organometallic Compounds−National Research Council (ICCOM-CNR), Via Madonna del Piano, 10, 50019 Sesto Fiorentino (FI), Italy; ⊥ Department of Civil Engineering, 19028 University of Salerno, Via Giovanni Paolo II, 132, 84084 Fisciano (SA), Italy; # Department of Industrial Engineering, University of Florence, Via di Santa Marta 3, Firenze 50139, Italy; ∇ Instituto de Tecnología Química Universitat Politècnica de València-Consejo Superior de Investigaciones Científicas, Universitat Politècnica de Valencia, Av. De los Naranjos s/n, 46022 Valencia, Spain

## Abstract

Formic acid (FA) is considered one of the most promising carriers of clean and safe dihydrogen. This study highlights the potential of using poly­(2,6-dimethyl-1,4-phenylene oxide) (PPO) as a support for AuNPs to produce H_2_ through formic acid dehydrogenation (FAD). The developed synthesis method allows for precise control over the gold content by completely encapsulating AuNPs (4–6 nm) within the PPO matrix, ensuring a uniform distribution of nanoparticles with an active cubic morphology. In an aqueous solution (or a water/DMAc mixture), the catalyst exhibited high activity, generating H_2_ without producing CO, underscoring its high selectivity for dehydrogenation. At 105 °C, the catalyst showed a TOF of 360 mol_FA_·mol_Au_^–1^·h^–1^ and an activation energy of 39.3 ± 2.6 kJ·mol^–1^. By optimizing the formic acid concentration and gold loading, the system achieved an impressive TOF of 600 mol_FA_·mol_Au_^–1^·h^–1^, comparable to the best values reported in the literature. Notably, the AuNPs-PPO system facilitates the FAD reaction without requiring additional bases or modified supports. The reaction order of 0.81 ± 0.04 with respect to FA concentration indicates the rapid diffusion of the reagent within the polymer matrix without limiting its reactivity. Lastly, the AuNPs-PPO catalytic system has been demonstrated to be reusable.

## Graphical abstract

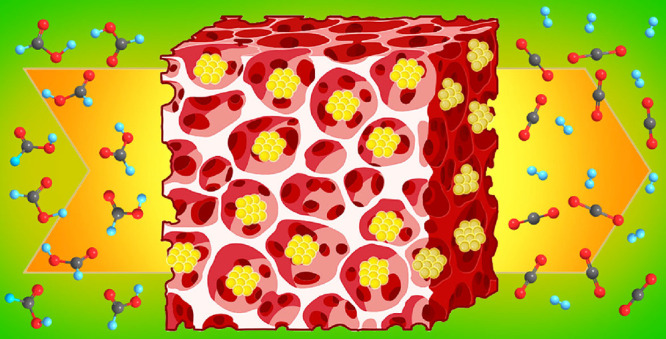

## Introduction

Hydrogen (H_2_) is a promising energy vector.^1,2^ H_2_ can potentially be sourced from biological materials and produced renewably through various processes, including water electrolysis, steam reforming of biomethane, and water-splitting reactions combined with solar technologies.^3–6^ Its combustion with oxygen releases water as a byproduct and generates 285.8 kJ/mol, which, due to its low molecular weight, corresponds to a calorific value of 141.8 MJ/kg.^7^ Conversely, due to its low density, high flammability, and reactivity, several challenges arise in the direct use of H_2_, particularly as a fuel for vehicles, such as cars, trucks, and trains. To achieve practical volumetric energy densities, H_2_ must be stored under high pressures (100–500 bar) or at cryogenic temperatures below −253 °C, resulting in densities of 7–27 and 70 kg/m^3^, respectively. By use of adsorbent materials at −196 °C and pressures ranging from 10 to 70 bar, densities between 20 and 50 kg/m^3^ can be achieved. At ambient pressure and temperature, hydrogen densities of 20–50 and 50–120 kg/m^3^ can be obtained using metal hydrides or hydrogen carrier molecules.^8^ These carriers offer high energy density and help mitigate or eliminate the safety risks associated with hydrogen’s flammability.

Ammonia,^9^ hydrazine,^10,11^ boranes,^12^ methanol,^13^ and formic acid^14,15^ are among the most promising molecules identified as hydrogen carriers. Alongside the sustainable production of hydrogen, a significant research challenge is the development of catalytic systems that enable the incorporation of hydrogen into carrier molecules, allowing for its release on demand for energy or chemical applications. Carbon dioxide (CO_2_) can be conveniently hydrogenated to produce formic acid,^16,17^ which can then be used as a hydrogen carrier.^14^ Formic acid is emerging as a promising H_2_ carrier due to its affordability, high hydrogen density, room temperature stability, and the ease and safety of storage and transportation. However, it also presents challenges, such as the formation of carbon monoxide in the dehydration pathway (Scheme 1), which poisons metal catalysts and limits their application in fuel cells. Despite these challenges, FA is a liquid at room temperature, nontoxic, cost-effective, can be produced from biomass, and presents high gravimetric (4.4 wt_H_2__%) and volumetric (53.4 g_H_2__/L) H_2_ content. It can release H_2_ under suitable catalytic conditions at mild temperatures, making it an ideal liquid organic hydrogen carrier (LOHC). Compared to other carriers, such as ammonia, borane, and methanol, formic acid effectively addresses the challenges related to the safe storage and transport of hydrogen. FAD enables the on-demand production of H_2_, making it particularly suitable for applications requiring intermittent hydrogen supply, such as portable fuel cells. FA can be synthesized via CO_2_ electroreduction powered by renewable energy sources, such as solar and wind, thereby creating a closed-loop system in which renewable hydrogen is generated, stored chemically in FA, and subsequently released through FAD for various applications. This process supports the transition to a more sustainable energy infrastructure by promoting the utilization of renewable hydrogen and reducing dependence on fossil fuels. FAD technology offers potential for powering portable electronic devices, such as smartphones, laptops, and electric vehicles, by delivering hydrogen for fuel cell operation. It can also serve as a reliable method for electricity generation in off-grid scenarios or as an emergency power backup. Moreover, formic acid serves as an efficient hydrogen carrier for fuel cell vehicles, contributing to the reduction of greenhouse gas emissions and fossil fuel consumption. Beyond energy storage and transport, FAD can supply hydrogen for various industrial chemical transformations. In a circular economy framework, CO_2_ emissions from industrial sources can be captured and converted into formic acid, which then acts as both a hydrogen carrier and a feedstock for producing valuable chemicals.^18^

**Scheme 1:**
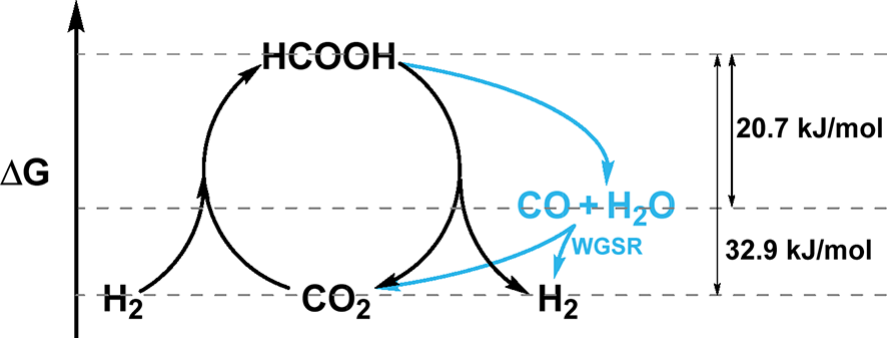
Δ*G*° Associated with Using Formic Acid as a Hydrogen Carrier

FA decomposition (FAD) can follow two pathways: dehydrogenation-producing H_2_ and CO_2_, and dehydration-producing CO and H_2_O (Scheme 1). The dehydration pathway is undesirable due to CO formation, which poisons metal catalysts and precludes application in fuel cells. The dehydrogenation pathway allows for reversible hydrogen storage, making FA an efficient hydrogen carrier.

While homogeneous catalysts offer high activity, they are challenging to separate, recover, and reuse and pose environmental concerns due to the use of toxic heavy metal ions for FAD. Heterogeneous catalysts are stable, recyclable, easily separable, and engineerable for specific reactors; however, they still face challenges in achieving high selectivity and catalytic activity comparable to those of homogeneous systems. Developing efficient heterogeneous catalysts remains a key research focus. At mild to moderate temperatures, noble metal-based nanocatalysts exhibit good activity and selectivity for FAD, whereas transition metals such as Ni and Fe show poor catalytic activity and selectivity.^18^ Catalyst design significantly influences FA decomposition efficiency, with research focusing on suppressing side reactions and improving hydrogen production and selectivity.

Typically, these metal nanoparticles are supported by inorganic compounds that offer good stability at high temperatures but do not allow fine regulation of the catalytic process. AuNPs have been widely employed in FAD exploring various inorganic supports such as zirconia, silica, titania, ceria, and carbonaceous materials, such as carbon black and graphene (see Table 2 and Discussion section).^19–26^ On the other hand, polymeric materials are optimal candidates as catalyst supports for the FAD process, which preferably must be carried out at low temperatures where selectivity toward H_2_ formation and the direct process of water gas shift reaction are favored, thus maximizing H_2_ production and avoiding the formation of CO (see Scheme 1). Polymeric supports enable fine-tuning of the chemical environment surrounding the catalytic sites.^27–29^ By selecting the specific polymer by chemical functionalization, polymer polarity can be precisely adjusted to control hydrophobicity/hydrophilicity, acidity/basicity, and the permeability to specific substrates. Their swelling with appropriate solvents can modulate the access of the reagents to the catalyst, thereby improving, for instance, the entry of organic compounds over others to direct or enhance the regio- and chemoselectivity of the catalytic system. As noteworthy examples, poly­(2,6-dimethyl-1,4-phenylene oxide) (PPO), the subject of this study as a polymeric support for AuNPs, and syndiotactic polystyrene (sPS) have been proven highly efficient in regulating the access of aromatic organic substrates to the catalytic sites while preventing the entry of water molecules—a property found helpful in the selective aerobic oxidation of primary alcohols to their corresponding aldehydes.^27,29–34^ PPO is a commercially available and inexpensive polymer material with enhanced thermal and chemical stability due to its high glass transition temperature (*T*_g_) and melting temperature (Tm), respectively, 215° and 245 °C.^35^ In addition, this polymer has recently been identified as nanoporous: the amorphous and crystalline phases of PPO are both highly permeable to small organic molecules such as solvents and reagents.^36,37^ The amorphous polymer can be crystallized by suitable solvent treatments, even leading to cocrystalline (CC) forms with small guest molecules.^37,38^ These features make PPO an interesting candidate as a support for heterogeneous catalysts, and here we report its application as a support for gold nanoparticles in the FAD to H_2_ reaction.

This study provides a proof of concept for the use of porous polymer-based supports to stabilize gold nanoparticles (AuNPs) for catalytic hydrogen production via FAD. The developed synthesis strategy enables precise incorporation of AuNPs within the PPO polymer matrix, resulting in well-dispersed, morphologically uniform particles. Initial catalytic tests demonstrate that the system effectively promotes hydrogen release from formic acid in aqueous or mixed solvent media, with high selectivity and minimal formation of undesired byproducts. Upon optimization of key variables, such as reactant concentration, temperature, and metal loading, the catalyst exhibits activity levels comparable to those of some of the most efficient systems reported in the literature, while operating under mild conditions and without requiring additional additives or modified supports. Moreover, the system exhibits promising stability and recyclability, underscoring its potential for practical applications in sustainable hydrogen generation.

## Experimental Section

### Materials

HAuCl_4_·3H_2_O (49 wt_Au_ %; Merck), poly­(2,6-dimethyl-1,4-phenylene oxide) (PPO, Sigma-Aldrich), sodium triethylborohydride (1.0 M in toluene; Merck), formic acid (98%; Pancreac), water (HPLC grade; Pancreac), potassium hydroxide (85%; Sigma-Aldrich), toluene (HPLC grade; Romil), *N*,*N*-dimethylacetamide (DMA, 99%, Merck), dimethyl sulfoxide (DMSO, 99,9%; Carlo Erba), 1-methyl-2-pirrolidinone, (99,5%, Merck), dioxane, (>99%, Merck), 1-butyl-3-methyl-imidazolium acetate (>95%; Merck), 1,3,5-trimethoxybenzene (99%, Merck), and deuterated solvents (Euriso-Top or Merck), unless otherwise stated, were used as received.

### Synthesis of AuNPs-PPO

The AuNPs-PPO catalyst was synthesized following a previously reported method.^30^ The following procedure was used to synthesize a catalyst with 2 wt % of gold. A 1 L round-bottomed three-necked flask equipped with a magnetic stirrer was charged with finely ground PPO (4.90 g) and anhydrous THF (800 mL). The mixture was stirred at room temperature under a nitrogen atmosphere for 24 h, followed by heating to the solvent’s reflux temperature for 1 h to ensure complete polymer swelling. Subsequently, HAuCl_4_·3H_2_O (0.200 g; 5.08 × 10^–4^ mol) was added at room temperature and stirred for 1 h. A solution of sodium triethylborohydride (4.1 mL, 1 M) in THF was then introduced at room temperature, causing an immediate color change from pale yellow to red. The resulting catalyst was quickly precipitated in methanol, collected by filtration, washed with fresh methanol, and dried under vacuum at room temperature.

### Instrumentation and Methods

Nuclear magnetic resonance (NMR) spectra were recorded with Bruker Avance spectrometers (600, 400, 300, and 250 MHz for ^1^H NMR). Chemical shifts were referred to tetramethylsilane as an external reference using the residual protio signals of deuterated solvents. Wide-angle X-ray diffraction (WAXD) patterns were obtained in reflection mode with an automatic Bruker D8 powder diffractometer using nickel-filtered CuK_α_ radiation. The gold content in AuNPs-PPO was measured by using inductively coupled plasma-optical emission spectrometry (ICP-OES) with an iCAP6000 spectrometer from Thermo Fisher Scientific. To prepare the sample, 50 mg of the AuNPs-PPO catalyst was digested in a Kjeldahl flask using 2.5 mL of sulfuric acid (98 wt %) at 250 °C for 30 min. After cooling to room temperature, 4.0 mL of hydrogen peroxide (30 wt %) was added. The suspension was reheated at 250 °C until a clear solution was obtained. Subsequently, 1.5 mL of freshly prepared aqua regia was added at room temperature, and the solution was diluted to a final volume of 10.0 mL with an aqueous solution of HCl (10 v%). Calibration was carried out using seven gold­(III) solutions of varying concentrations, prepared by serial dilution of a standard solution (1.000 ± 0.002 g/L in water with 2 wt % HCl) using water and a 10 vol % aqueous HCl solution. Electron microscopy characterization was performed by using a Thermo Fisher TALOS F200X G2 microscope. High-resolution transmission electron microscopy (HR-TEM) imaging and scanning transmission electron microscope energy-dispersive X-ray spectroscopy (STEM-EDX) mapping were performed using a beam acceleration of 200 kV. The gold *d*-spacing was identified by comparison with the cubic Au (1744486) entry from the Cambridge Crystallographic Data Centre (CCDC) database. The dimensional distribution of the particles was assessed by considering one hundred entities from TEM and STEM images relative to different spots on the sample. When the particle was recognized as elongated rather than spherical, a larger diagonal value was acquired for the estimate. H_2_ and CO_2_ were identified and quantified with a Trace1300 gas chromatograph coupled with a thermal conductivity detector (GC-TCD) and a TG-BOND Q column (30 m x 0.32 mm x 10 μm from Thermo Scientific, USA) injecting 100 μL samples drawn with a Hamilton Gastight syringe (Hamilton Co., USA).

### General Procedure for Formic Acid Decompositions Catalyzed by AuNPs-PPO (Referred to Entry 1 of Table 1)

A 25 mL round-bottomed flask equipped with a magnetic stir bar and a refrigerant was charged with formic acid (0.470 g, 0.01 mol), water (4.0 mL), and AuNPs-PPO (100 mg; 1 × 10^–5^ mol_Au_). The mixture was stirred for 24 h at 100 °C.

The flask was cooled at room temperature, and a solution of 1,3,5-trimethoxybenzene (0.187 g, 1 mmol, in 4 mL of methanol) was added as an internal standard. The polymer was separated by centrifugation, and the filtrate was analyzed by ^1^H NMR spectroscopy using methanol-*d*_4_ as a solvent, determining a yield of 78% (see Figure S1 of the Supporting Information). Calibrating the signal relative to the peak of the standard at 100, the equation used for the calculation of FA conversion is as follows:

Conversion(%)=100−FAintegralvalue

The reaction was performed in a pressure vessel equipped with a magnetic stir bar under the same conditions. After 24 h, 100 μL from the reaction mixture was withdrawn with a Hamilton Gastight syringe and analyzed by GC-TCD. H_2_ and CO_2_ were the unique products observed. (Supporting Information, Figure S2).

**Table 1:** Decomposition of Formic Acid to Hydrogen and Carbon Dioxide Promoted by AuNPs-PPO

entry	solvent	*T* (°C)	FA/Au (mol ratio)	base	base/FA (mol ratio)	*t* (h)	conv.^a^ (%)	TOF^b^ (mol_FA_·mol_Au_^–1^·h^–1^)
1	H_2_O	100	1000			24	78	32^c^
2	H_2_O/Tol	100	1000			24	0	0
3	H_2_O/1,4-D	100	1000			24	28	11,7
4	H_2_O/NMP	100	1000			24	65	27
5	H_2_O/DMAc	100	1000			24	83	35
6	H_2_O/[BMI]Ac	100	1000			24	67	28,3
7	H_2_O/DMSO	100	1000			24	15	6,2
8	H_2_O	50	1000			24	11	4,5
9	H_2_O	60	1000			24	51	21
10	H_2_O	80	1000			1 [24]	15.3 [68]	153 [28]
11	H_2_O	90	1000			1	22.5	225
12	H_2_O	100	1000			1	33.3	330
13	H_2_O	105 (reflux)	1000			1	36	360
14	H_2_O	105 (reflux)	500			1	39	195
15	H_2_O	105 (reflux)	2000			1	30	600
16	H_2_O	60	1000	KOH	(0.5:1)	24	19	7,9
17	H_2_O	60	1000	KOH	1:1	24	24	10
18	H_2_O	60	1000	KOH	3:1	24	0	0
19	H_2_O	60	1000	NEt_3_	1:1	24	0	0

a Reaction condition: AuNPs-PPO (100 mg; Au = 1 × 10^–5^ mol), solvent (4 mL).

b Determined by ^1^H NMR spectroscopy using 1,3,5-TMB as an internal standard.

c Turnover frequency: mol of reacted FA·mol_Au_^–1^·time^–1^.

### General Procedure for Catalyst Recycling Tests

A 25 mL two-necked round-bottom flask equipped with a magnetic stir bar and a refrigerant was charged with formic acid (0.0470 g, 1 mmol), water (4 mL), and AuNPs-PPO (100 mg). The mixture was stirred for 45 min at 80 °C. An aliquot of the reaction mixture was analyzed by ^1^H NMR, adding 0.5 mL of a solution of 1,3,5-trimethoxybenzene (0.462 g in 2.5 mL of MeOD-*d*_4_) as an internal standard. The reaction mixture was analyzed by ^1^H NMR spectroscopy. After the consumption of formic acid, it was added to the flask for four more catalytic cycles. At each recycle test, formic acid consumption was quantitatively confirmed by ^1^H NMR using the internal standard method.

## Results

### Synthesis and Characterization of the AuNPs-PPO Catalyst

The AuNPs-PPO catalytic system was prepared according to a recently developed procedure.^30^ The procedure allowed for fine control of the gold content. A catalyst with 2 wt % gold was prepared and investigated in the FAD reaction. The synthetic procedure involved dissolving the polymer in tetrahydrofuran (THF), adding tetrachloroauric acid as a gold precursor, reducing it with sodium triethylborohydride, and rapidly coagulating the polymer with embedded AuNPs in a large amount of methanol. The gold content was confirmed by inductively coupled plasma-optical emission spectroscopy (ICP-OES) analysis of the acidic mineralized catalyst.

The procedure ensures the formation of small nanoparticles of 4–6 nm, as verified by wide-angle X-ray diffraction (WAXD, Figure 1a) analysis, determining the size of the Au crystallites by the Scherrer equation (using the width at full width at half-maximum, fwhm, and the position of the ⟨111⟩ reflex of the face-centered cubic, fcc, gold at 2θ = 38°).^39^ The polymer support was found in amorphous form, as observable from the 2θ range 5–35° of the WAXD diffraction pattern in Figure 1a. X-ray photoelectron spectroscopy (XPS) measurements were carried out using a nonmonochromated Mg–Kα X-ray source (1253.6 eV) and a VSW HAC 5000 hemispherical electron energy analyzer.

**Figure 1:**
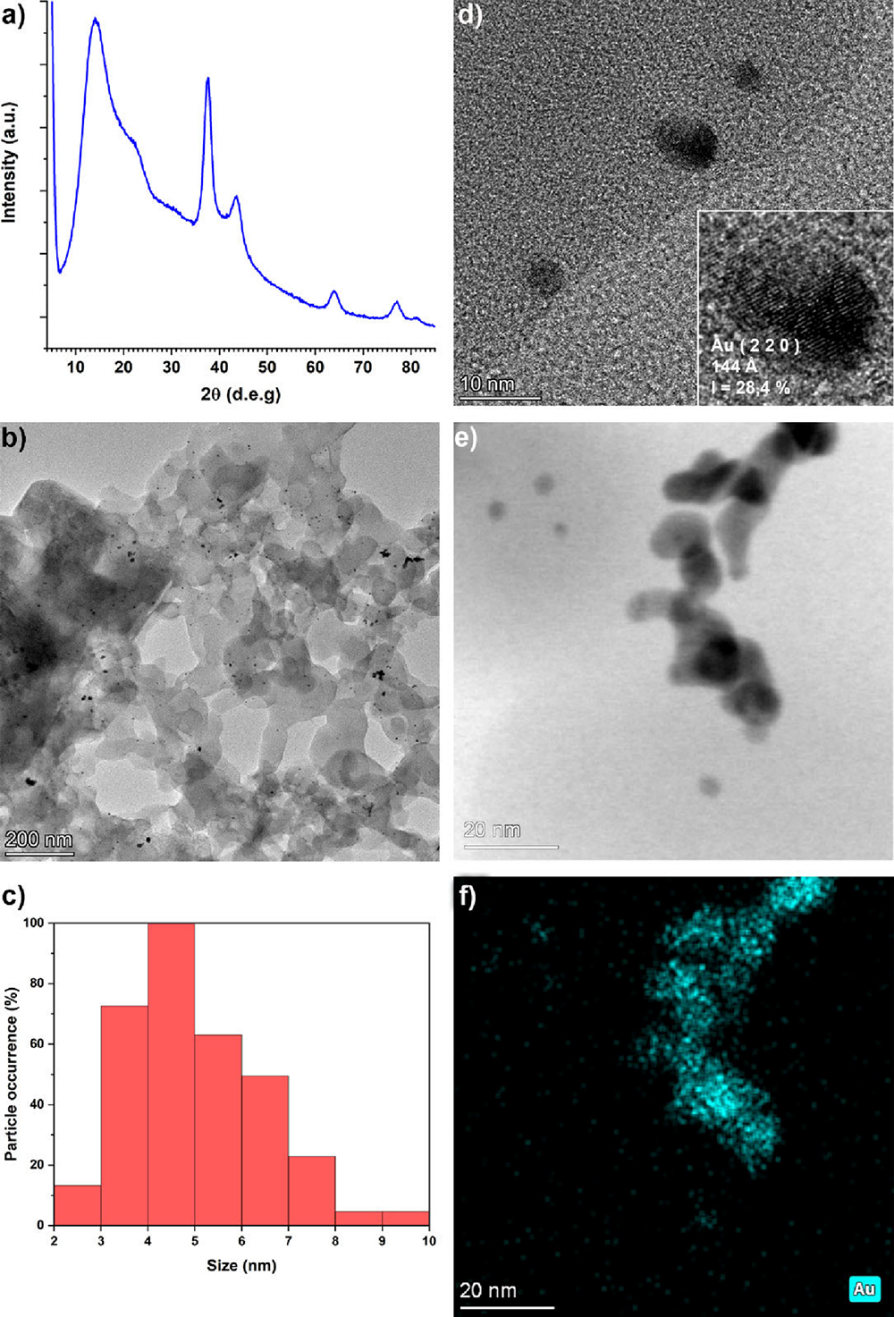
Characterization of AuNPs-PPO: WAXD pattern (a); TEM micrograph (b) with the corresponding size distribution plot (c); HR-TEM of nanoparticles (d) with inset showing a detail of AuNPs with a ⟨220⟩-plane exposed); bright-field STEM image (e) with the corresponding EDX micrograph highlighting the gold distribution (f).

The synthetic protocol also affords an optimal dispersion of the AuNPs in the polymer matrix. From the high-resolution transmission electron microscopy (HR-TEM) examination, the nanoparticles have a size in agreement with that determined by WAXD analysis and are generally well-dispersed in the polymer support, with some local aggregates of AuNPs (Figure 1b,c). The consistency of these aggregates of small gold nanoparticles was further verified by bright-field scanning transmission electron microscopy (BF-STEM) analysis coupled with energy-dispersive X-ray (EDX) spectroscopy analysis (Figure 1d,e). The estimation of the dimensional distribution of the particles highlighted that most particles have a dimension between 3 and 6 nm. Interestingly, the procedure resulted in the formation of spherical nanoparticles with diameters below 4 nm. It was, however, possible to notice by TEM on the particles the ubiquitous presence of the lattice fringes related to cubic Au (like ⟨220⟩ planes of the fcc gold in the inset in Figure 1c).^40,41^ X-ray photoelectron spectroscopy (XPS) analysis of the AuNPs-PPO catalyst further confirmed the presence of gold (Figure S22).

### Catalytic Tests of FAD Promoted by AuNPs-PPO

AuNPs-PPO (2 wt % Au, 100 mg, 1 × 10^–5^ mol Au) was tested as a catalyst in the decomposition reaction of formic acid in the form of finely ground powders obtained by simply grinding the crude catalyst in a mortar. The tests were carried out in batches in a round-bottom flask, thermostated at the desired temperature, equipped with a magnetic stir bar and an open condenser to vent the evolved gases into the air. The tests were initially conducted by exploring the effect of the solvent at a temperature of 100 °C, using an FA/Au mol ratio of 1000, corresponding to a gold loading of 0.1 mol%, for 24 h and in the absence of other additives. The catalytic test conducted in water afforded a conversion of 78%, corresponding to a turnover frequency (TOF) of 32 mol_FA_·mol_Au_^–1^·h^–1^ (entry 1 of Table 1 and Figure 2). The corresponding blank tests performed in the absence of AuNPs-PPO or in the presence of PPO alone did not produce FA conversion.

**Figure 2:**
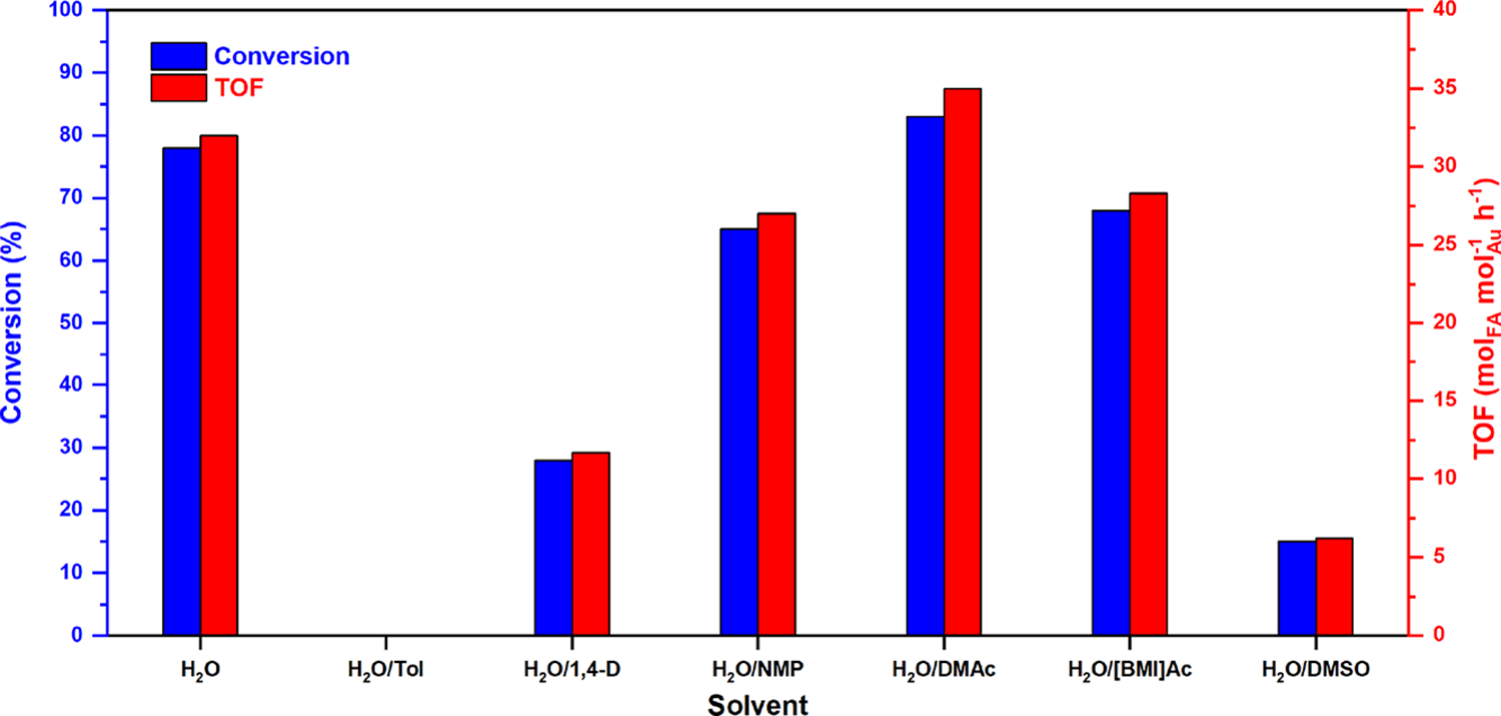
Effect of the solvent on the decomposition of formic acid to hydrogen promoted by AuNPs-PPO (entries 1–7, Table 1).

Tests carried out in stainless steel pressure reactors both in the presence and in the absence of water, i.e., using FA neat, when analyzed by gas chromatography, demonstrated the exclusive formation of H_2_ and CO_2_ (see Figure S2) and therefore excluded the formation of CO for the AuNPs-PPO catalyst.

Subsequently, the effect of introducing a cosolvent in a 1:1 v/v ratio relative to water was evaluated while maintaining a total volume of 4 mL for the cosolvent/water mixture (entries 2–7 of Table 1 and Figure 2). The use of a nonpolar cosolvent, such as toluene, led to a complete loss of catalytic activity (entry 2). As the polarity of the cosolvent increased, a gradual enhancement in catalytic activity was observed, progressing from 1,4-dioxane (1,4-D; entry 3) to *N*-methyl-2-pyrrolidone (NMP; entry 4), and then to dimethylacetamide (DMAc; entry 5). The highest conversion of 83%, associated with a TOF of 35 mol_FA_·mol_Au_^–1^·h^–1^, was achieved with the H_2_O/DMAc mixture. However, further increases in cosolvent polarity, utilizing the ionic liquid 1-butyl-3-methyl-imidazolium acetate [BMIm]Ac or dimethyl sulfoxide (DMSO), did not yield any further enhancement in conversion (entries 6 and 7).

Temperature has a significant effect on FAD. Nevertheless, the catalytic system showed modest activity, even at low temperatures of 50–60 °C (entries 8 and 9), providing FA conversions to H_2_ of 11 and 51%, respectively, with corresponding TOF values of 4.5 and 21 mol_FA_·mol_Au_^–1^·h^–1^.

In order to better evaluate the effect of temperature and to estimate the activation energy associated with the FAD reaction promoted by AuNPs-PPO, the subsequent tests were carried out at reaction times of 1 h in the range 80–105 °C (the reaction temperature of 105 °C was measured inside the reactor by heating the reactor to the reflux of the FA/H_2_O reaction mixture) (entries 10–13). From the graph in Figure 3a, it is possible to observe an almost linear increase in the conversion and catalytic activity with temperature, reaching an excellent TOF of 360 mol_FA_·mol_Au_^–1^·h^–1^ at 105 °C. The corresponding Arrhenius plot (ln­(TOF) vs 1/T; Figure 3b) allowed to determine an activation energy of 39.3 ± 2.6 kJ·mol^–1^ (9.4 ± 0.6 kcal·mol^–1^).

**Figure 3:**
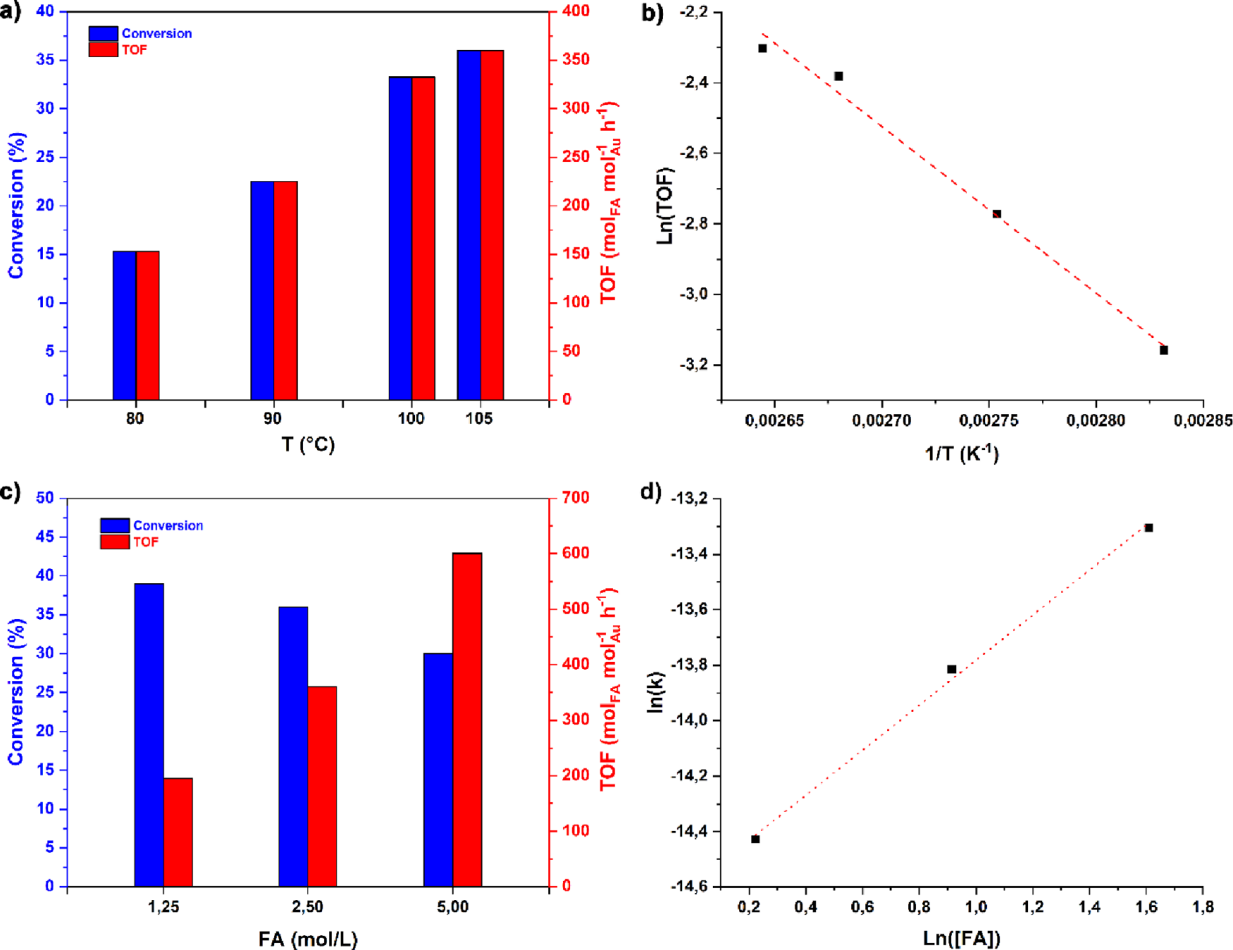
Decomposition of formic acid to hydrogen promoted by AuNPs-PPO: effect of the reaction temperature (graph a) with the corresponding Arrhenius plot (graph b) for the determination of the activation energy (entries 10–13, Table 1); effect of the FA concentration (graph c) and the graph ln­(k) vs ln­([FA]) (graph d) for the determination of the reaction order w.r.t. FA concentration (entries 13–15, Table 1).

The molar ratio between FA and Au also significantly affected the reaction (entries 13–15). As shown in the graph in Figure 3c, the catalytic activity increases notably when the FA/Au molar ratio is raised to 2000 (with an Au loading of 0.05 mol % and [FA] = 5 mol·L^–1^), achieving an impressive TOF of 600 molFA·molAu^–1^·h^–1^. The corresponding ln­(*k*_app_) vs ln­([FA]) graph allowed us to determine a reaction order of 0.81 ± 0.04 with respect to the FA concentration. Additionally, to better assess the influence of a base additive on the reaction, tests were conducted at a lower temperature of 60 °C. However, the introduction of potassium hydroxide at molar ratios to FA of 0.5:1, 1:1, and 3:1 (entries 16–18), as well as triethylamine (entry 19), did not enhance conversion compared to the test performed in neat water as the solvent (entry 9) (Figure 4).

**Figure 4:**
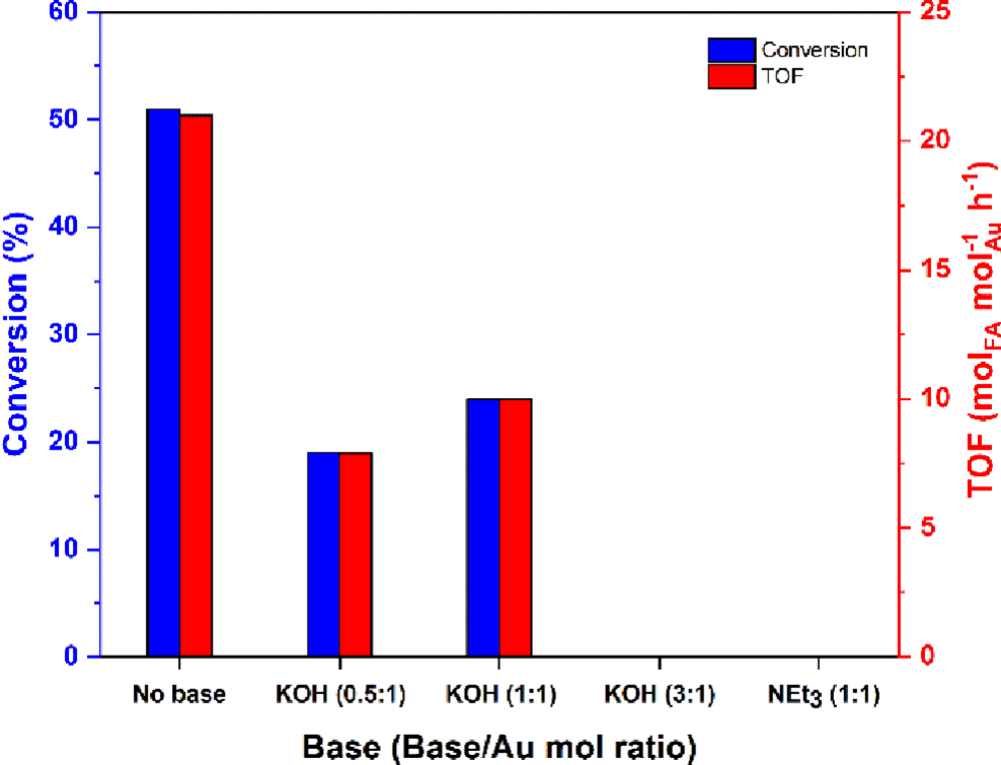
Effect of the base additives on the decomposition of formic acid to hydrogen promoted by AuNPs-PPO (entries 9 and 16–19, Table 1).

Finally, the recyclability of the catalytic system was evaluated. The catalyst was utilized for five consecutive tests in neat water at an FA/Au molar ratio of 100 and 80 °C. As shown in the graph in Figure 5a, both conversion and activity remained consistent throughout the reuse tests of the catalyst. To further support these findings, the catalyst used in these tests was analyzed by WAXD both before and after the repeated catalysis experiments. The resulting patterns perfectly coincide, indicating that the nanoparticles do not undergo coalescence or significant structural changes during the five repeated tests conducted.

**Figure 5:**
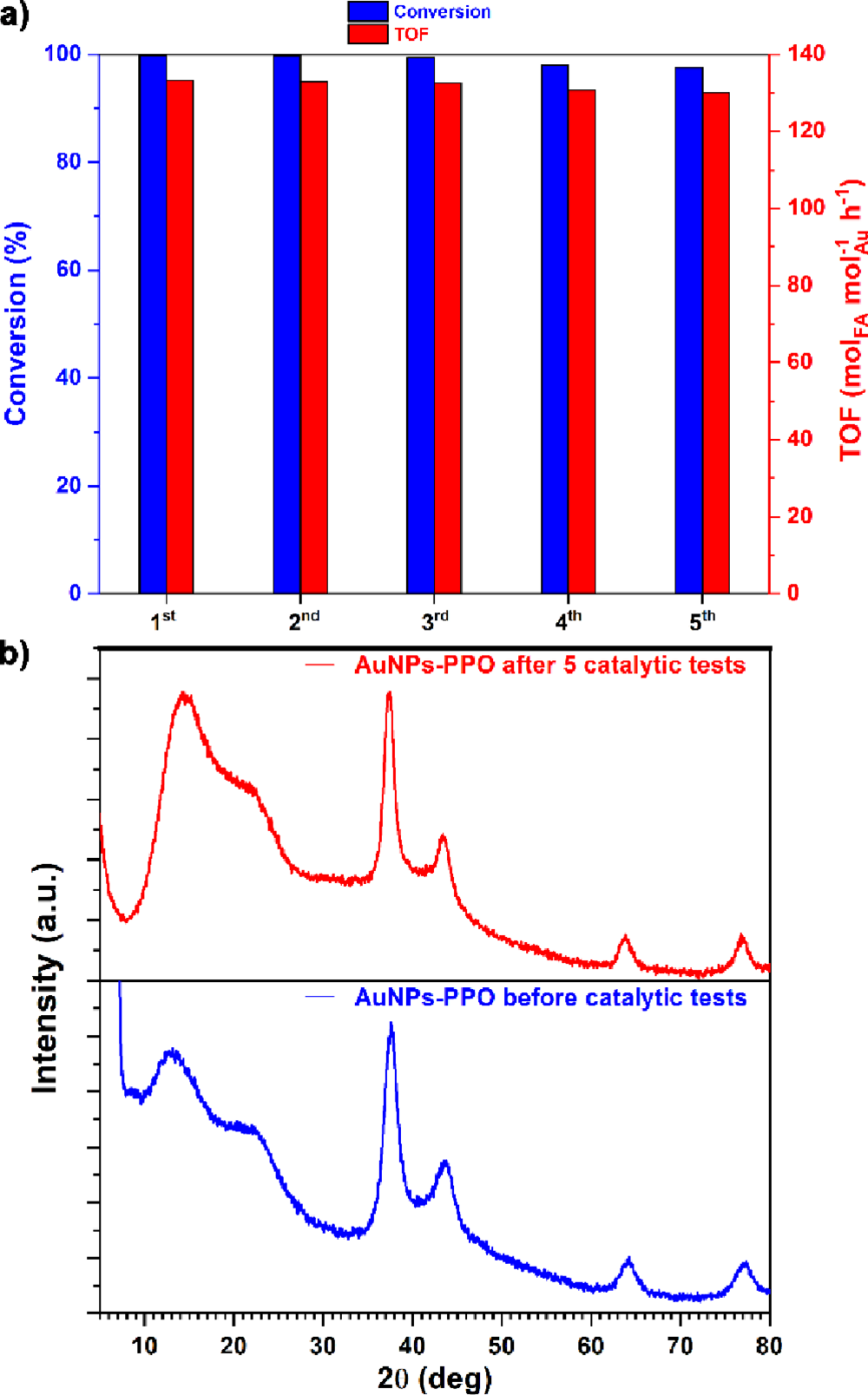
Repeated recycle tests of AuNPs-PPO in formic acid decomposition to hydrogen (graph a; reaction conditions: FA = 1 mmol; H_2_O = 4 mL; FA/Au = 100/1; 45 min; 80 °C); WAXD patterns of AuNPs-PPO before and after five catalytic tests (b).

## Discussion

The synthetic procedure used to support AuNPs on PPO allows for easy regulation of the gold content by ensuring the complete trapping of AuNPs within the polymer matrix during solution synthesis and precipitation in methanol. The resulting nanoparticles exhibit a size of 4–6 nm, are uniformly distributed within the polymer matrix, and display a cubic geometry, as confirmed by high-resolution TEM analysis. This analysis reveals the presence of fcc gold 220 planes, crystalline facets known for their high activity in catalytic applications due to the metal’s low coordination number (Figure 1). However, it is recognized that very small, subnanometric nanoparticles demonstrate enhanced catalytic activity in FAD applications.^22,42–44^ The synthetic strategy here adopted was used in previous studies using the nanoporous syndiotactic polystyrene (sPS) and its copolymers with cis-1,4-polybutadiene (sPSB) as the support for AuNPs, producing similar results in terms of the formation of nanoparticles of a few nanometers in size.^27,29,31–34^

Preliminary FAD tests conducted with AuNPs-PPO demonstrated that the catalytic system can be effectively tuned in the presence of different solvents (see entries 1–7 of Table 1 and Figure 2). The catalyst proved to be highly effective in neat water; however, the introduction of highly apolar solvents, which can dissolve the polymeric support, was found to be unfavorable. The best performance was observed with the H_2_O/DMAc mixture (entry 5, Table 1). DMAc is capable of swelling PPO without dissolving it, thereby enhancing access to the catalytic sites for FA.

The subsequent tests were performed in pure water to ensure cost efficiency and process sustainability. Water is commonly used in FAD catalytic tests not only as a solvent but also as a crucial additive that helps prevent the decomposition of FA into CO and H_2_O, thus shifting the equilibrium back toward the reagent (see Scheme 1). In the case of the AuNPs-PPO catalytic system, the analysis of the gases evolved during catalysis revealed no formation of CO (Figure S2). This finding highlights the catalyst’s high selectivity in promoting the dehydrogenation reaction over the dehydration of FA.

As expected, the temperature significantly affected the catalytic activity, reaching a TOF of 360 mol_FA_·mol_Au_^–1^·h^–1^ at 105 °C (entry 13, Table 1). The activation energy determined over the 80–105 °C temperature range was 39.3 ± 2.6 kJ·mol^–1^ (see entries 10–13, Table 1 and Figure 3a,b), which is in line with that reported for the most active gold-based catalytic systems and bimetallic gold–palladium or gold–platinum nanoparticles, typically found in the range of 13–55 kJ·mol^–1^.^45–48^

By investigation of the effect of FA concentration and gold loading, a remarkable TOF value of 600 mol_FA_·mol_Au_^–1^·h^–1^ was achieved (entry 15 of Table 1 and Figure 3c,d). This value competes with the highest activity values reported in the literature for the gold-based catalysts listed in Table 2. It is interesting to note that all these catalytic systems previously reported reach their maximum catalytic activity in the presence of bases as cocatalysts^22,49–51^ or when using supports modified with Schiff bases^52^ or amino^53^ groups. The use of additional inorganic alkali, such as NaOH or KOH, external bases like amines (Table 2), or buffer conditions, to enhance catalytic activity presents challenges for industrial scale-up and automotive applications. Notably, the AuNPs-PPO catalyst achieved a preliminary TOF value of 600 mol_FA_·mol_Au_^–1^·h^–1^ without the need for additional bases or modifications to the support, using neat water as the solvent (entry 15 of Table 1). In many homogeneous catalytic systems, especially those based on noble metals such as Ru, Ir, Rh, or Pd, the presence of external bases (e.g., NaOH, KOH, NEt_3_, and NaHCO_3_) is often required to achieve acceptable rates and selectivity.^54^ This imposes serious penalties in terms of scalability, safety, system complexity, and sustainability. Strong bases are hazardous and corrosive, complicating safe handling and reactor materials, especially critical in automotive systems.^55^ Alkaline conditions can lead to catalyst deactivation through ligand degradation or metal precipitation, reducing the long-term performance. The use of a stoichiometric base produces saline waste streams, undermining environmental sustainability and increasing post-treatment costs. Reagent costs and added process steps reduce the economic feasibility of large-scale or mobile hydrogen production from formic acid.^56,57^

**Table 2:** Gold Nanoparticles-Based Catalysts for FAD

catalyst	*T* (°C)	additive	TOF (h-1)	reference
AuNPs/NH_*2*_-C	125		2016	^53^
Au/NdZrO_2_	50	NEt_3_	2452	^50^
Au/CeZrO_2_	50	NEt_3_	2154	^50^
Au/SmZrO_2_	50	NEt_3_	1906	^50^
Au/ZrO_2_	50	NEt_3_	1525	^50^
Au@SiO_2_	130	HCOOH/Ar	958	^51^
Au@Schiff-base-SiO_2_	50		2882	^52^
Au/ZrO_2_	50	NEt_3_	1590	^22^
Au/ZrO_2_	60	dimethylethanolamine	2015	^49^

The role of the polymer support was investigated by determining the reaction order with respect to the FA by varying its concentration in the range of 1.25- 5.00 mol/L. The plot of ln­(k) vs ln­([FA]) determined a reaction order w.r.t. [FA] of 0.81 ± 0.04 (entries 13–15 of Table 1 and Figure 3c,d). This value rules out an FAD reaction controlled by the diffusion of FA in the polymer matrix, which would have resulted in a zero-order reaction. However, the value is not exactly 1, indicating that diffusion of the reagent still plays a role in determining the catalytic activity. This suggests the possibility of further improving the catalytic system by enhancing its porosity and the access of catalytic sites to the reagents. Additionally, the size of the metal particles needs to be improved. The 4–6 nm size found for the AuNPs-PPO system can still be improved, and the synthetic procedure must be refined in order to obtain subnanometric particles, which have been found to be highly efficient in previous studies reported in scientific literature. These results can pave the way for increasingly efficient systems by progressively refining control over the porosity of the matrix and the size of the nanoparticles. Finally, the AuNPs-PPO catalytic system has demonstrated excellent reusability (see Figure 5).

## Conclusions

This study serves as a proof of concept for demonstrating the applicability of PPO as a support for AuNPs in producing H_2_ through formic acid dehydrogenation. The synthetic approach developed for synthesizing the AuNPs-PPO catalytic system allows precise control over the gold content by fully trapping AuNPs, sized 4–6 nm, within the PPO matrix during the synthesis, resulting in uniformly distributed particles with active AuNPs displaying a cubic morphology. Preliminary FA dehydrogenation tests indicated that the catalyst operates very effectively in water (or a water/DMAc mixture) without generating CO, indicating high dehydrogenation selectivity and leading to H_2_ production. Temperature studies revealed that increasing the reaction temperature to 105 °C results in a TOF of 360 mol_FA_·mol_Au_^–1^·h^–1^ and an activation energy of 39.3 ± 2.6 kJ·mol^–1^. By optimizing the FA concentration and gold loading, the system achieved an impressive TOF of 600 mol_FA_·mol_Au_^–1^·h^–1^, which is competitive compared to the best literature values, without needing additional bases or modified supports. Analysis of the reaction order (0.81 ± 0.04) with respect to FA concentration suggests that the diffusion of FA in the polymer matrix to reach the catalytic sites does not limit FA reactivity. These results can pave the way for increasingly efficient systems by progressively refining the control over the porosity of the matrix and the size of the nanoparticles. Further enhancement could be achieved by improving support porosity and reducing nanoparticle size. Finally, the AuNPs-PPO catalytic system exhibited excellent reusability.

## Supplementary Material



## Notes

Additional information on the NMR, GC, and XPS characterizations (PDF)
