# Supplementary material for: Hydrogen Production from Formic Acid Decomposition Promoted by Gold Nanoparticles Supported on a Porous Polymer Matrix
Source: Energy Fuels. 2025 Jul 10;39(29):14320–9. doi: 10.1021/acs.energyfuels.5c01537 (PMC12308819; doi:10.1021/acs.energyfuels.5c01537)
Supplement: Supplementary file 1 [file ef5c01537_si_001.pdf]

Supporting information

Hydrogen Production from Formic Acid Decomposition Promoted by Gold Nanoparticles Supported on Porous Polymer Matrix

*Matteo Diglio[a‡], Irene Contento[a,b‡], Salvatore Impemba[a,b], Enrico Berretti[c], Paolo Della Sala[a], Giuseppina Oliva[d], Vincenzo Naddeo[d], Stefano Caporali,[e] Ana Primo,[f] Carmen Talotta[a], Carmine Gaeta[a], Carmine Capacchione[a,b], Alfonso Grassi[a,b] and Antonio Buonerba[a,b]**

[a] Department of Chemistry and Biology *"Adolfo Zambelli"*, University of Salerno, Via Giovanni Paolo II, 132, 84084, Fisciano (SA), Italy.

[b] Interuniversity Consortium of Chemical Reactivity and Catalysis (CIRCC), Via Celso Ulpiani, 27, 70126 Bari, Italy, and NANO_MATES (Research Centre for Nanomaterials and Nanotechnology, Via Giovanni Paolo II, 132, 84084, Fisciano (SA), Italy.

[c] Institute of Chemistry of the Organometallic Compounds – National Research Council (ICCOM-CNR), Via Madonna del Piano, 10, 50019 Sesto Fiorentino (FI), Italy.

[d] Department of Civil Engineering, University of Salerno, Via Giovanni Paolo II, 132, 84084, Fisciano (SA), Italy.

[e] Department of Industrial Engineering, University of Florence, Via di Santa Marta 3, Firenze 50139, Italy

[f] Instituto de Tecnología Química Universitat Politècnica de València-Consejo Superior de Investigaciones Científicas, Universitat Politècnica de Valencia, Av. De los Naranjos s/n, 46022 Valencia, Spain

Supporting information

‡ These authors equally contributed.

* Corresponding author: A. Buonerba (abuonerba@unisa.it).

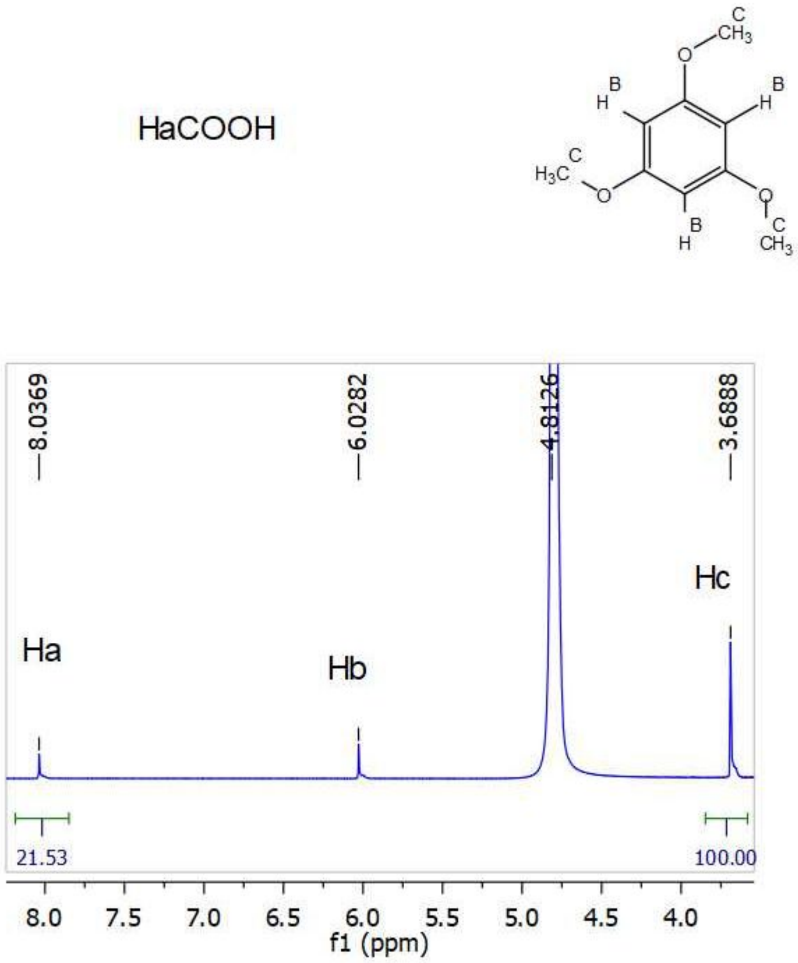

Figure S1. ^{1}H NMR spectrum (400 MHz, methanol-d_4) of a catalytic test of formic acid decomposition promoted by AuNPs-PPO (entry **1**, Table 1).

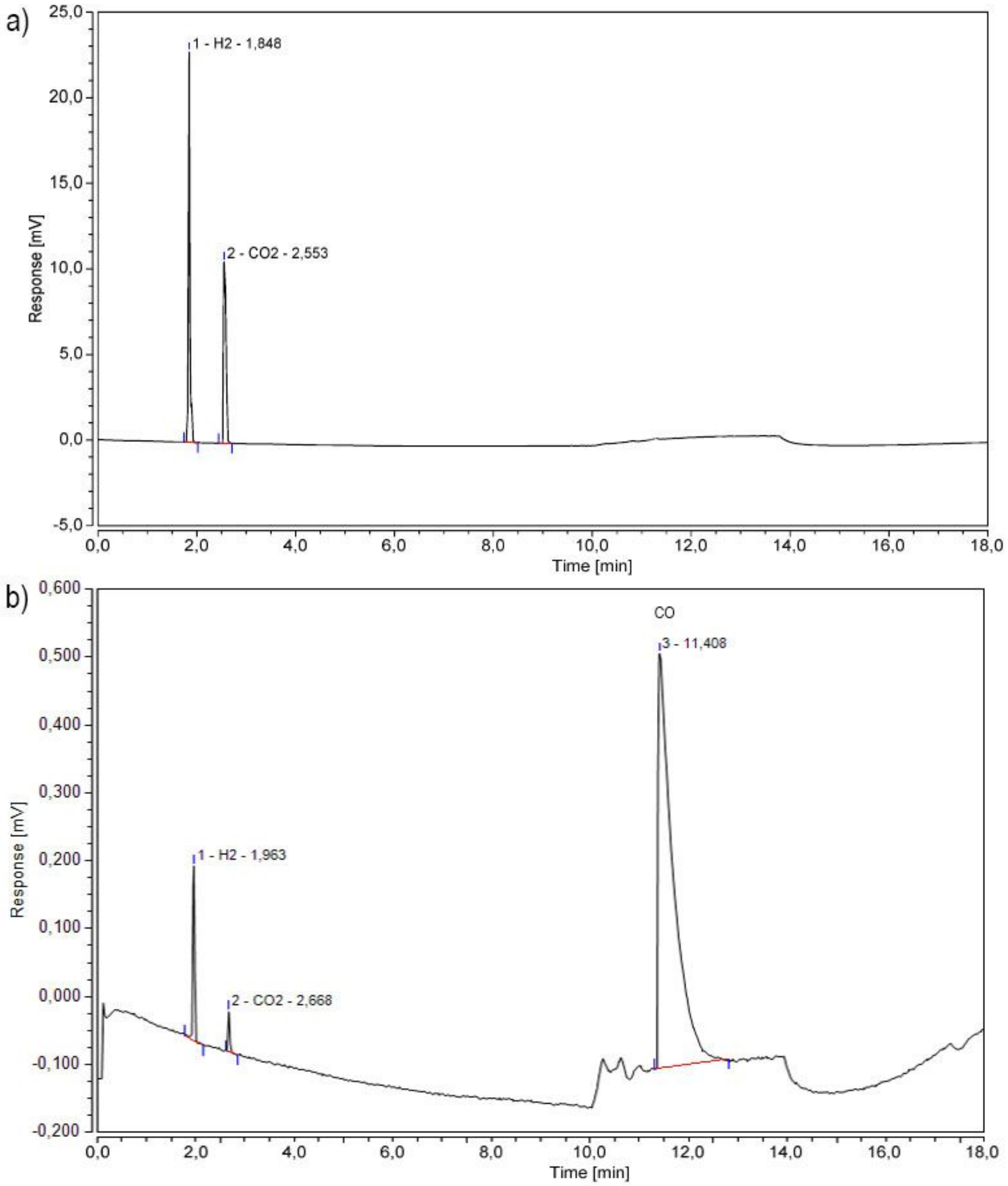

Figure S2. Gas chromatograms of: a) catalytic test of formic acid decomposition promoted by AuNPs-PPO (entry **1**, Table 1); b) H_2, CO_2 and CO mixture.

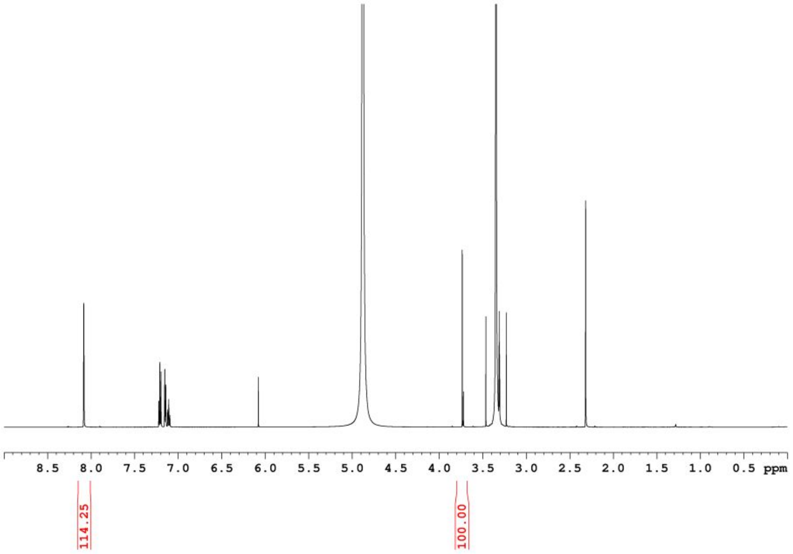

Figure S3. ^{1}H NMR spectrum (400 MHz, methanol-d_4) of a catalytic test of formic acid decomposition promoted by AuNPs-PPO (entry **2**, Table 1).

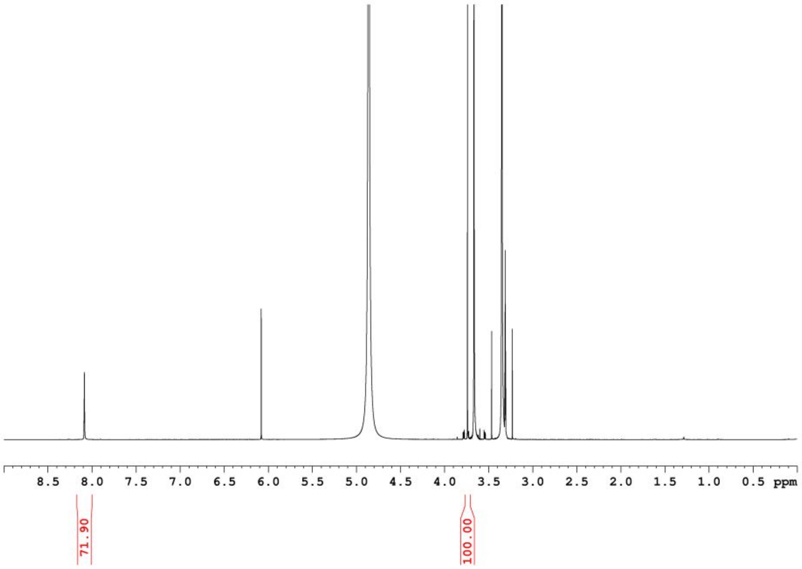

Figure S4. ^{1}H NMR spectrum (400 MHz, methanol-d_4) of a catalytic test of formic acid decomposition promoted by AuNPs-PPO (entry **3**, Table 1).

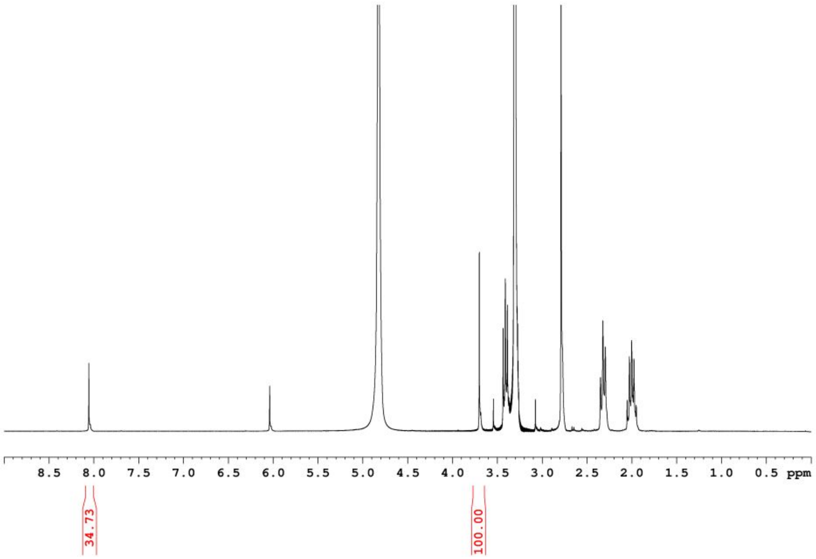

Figure S5. ^{1}H NMR spectrum (400 MHz, methanol-d_4) of a catalytic test of formic acid decomposition promoted by AuNPs-PPO (entry **4**, Table 1).

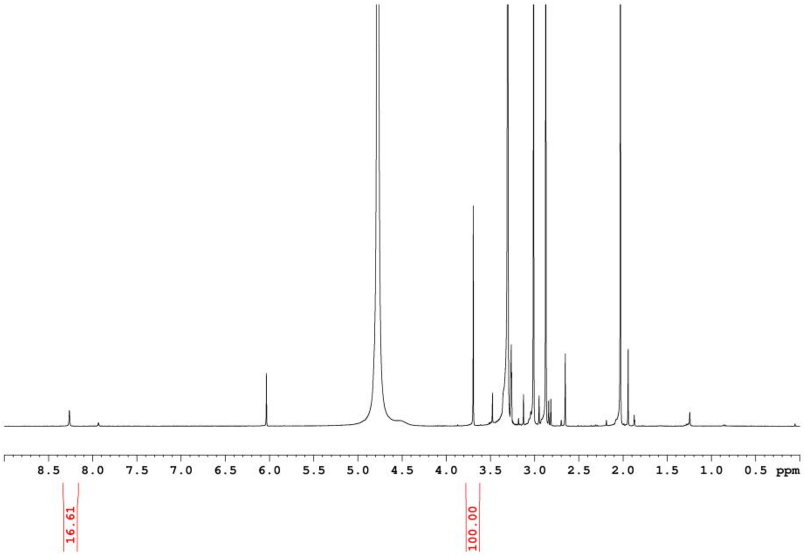

Figure S6. ^{1}H NMR spectrum (400 MHz, methanol-d_4) of a catalytic test of formic acid decomposition promoted by AuNPs-PPO (entry **5**, Table 1).

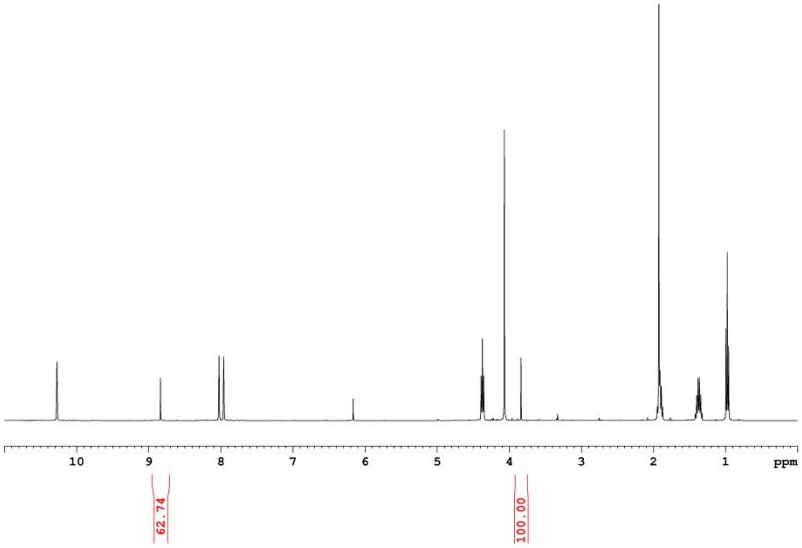

Figure S7. ^{1}H NMR spectrum (400 MHz, acetonitrile-d_3) of a catalytic test of formic acid decomposition promoted by AuNPs-PPO (entry **6**, Table 1).

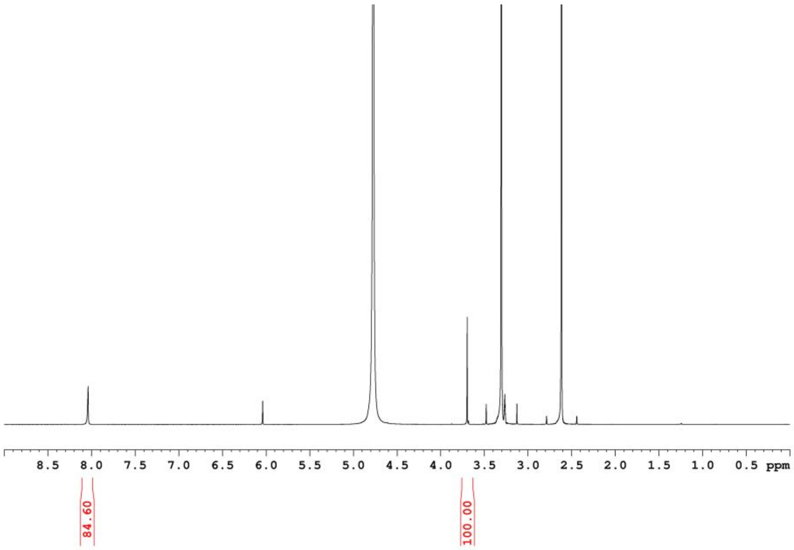

Figure S8. ^{1}H NMR spectrum (400 MHz, methanol-d_4) of a catalytic test of formic acid decomposition promoted by AuNPs-PPO (entry **7**, Table 1).

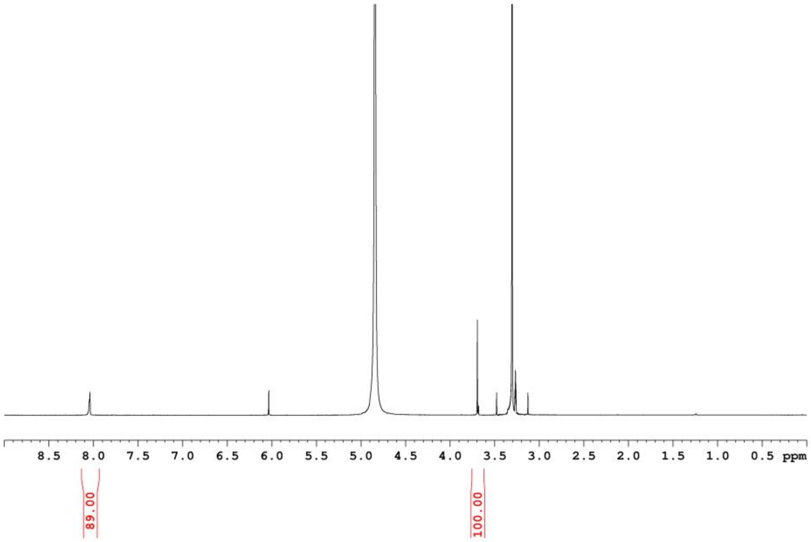

Figure S9. ^{1}H NMR spectrum (400 MHz, methanol-d_4) of a catalytic test of formic acid decomposition promoted by AuNPs-PPO (entry **8**, Table 1).

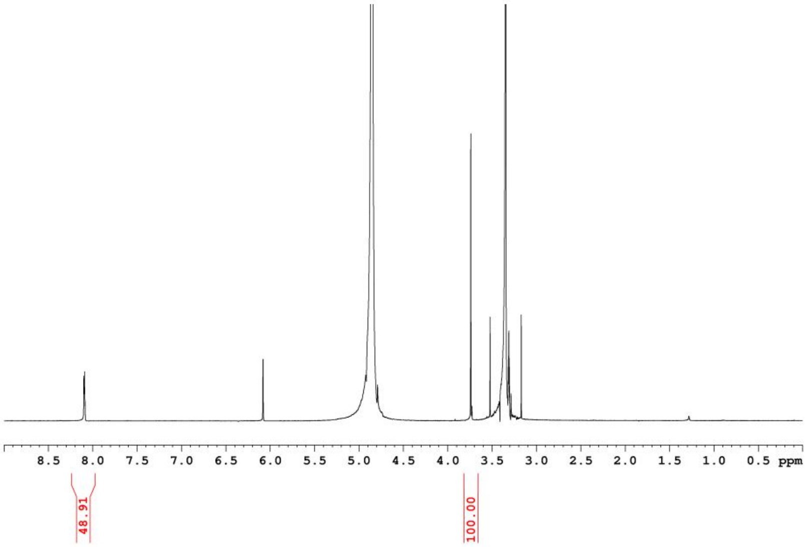

Figure S10. ^{1}H NMR spectrum (400 MHz, methanol-d_4) of a catalytic test of formic acid decomposition promoted by AuNPs-PPO (entry **9**, Table 1).

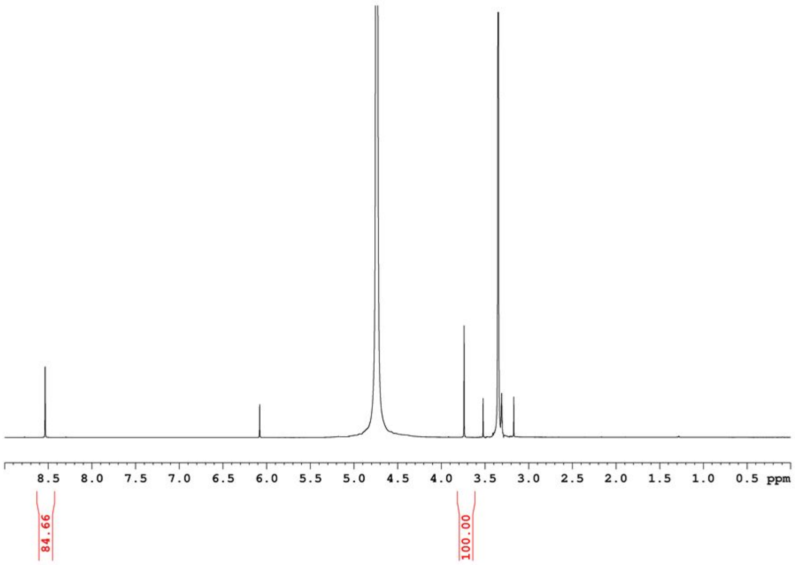

Figure S11. ^{1}H NMR spectrum (400 MHz, methanol-d_4) of a catalytic test of formic acid decomposition promoted by AuNPs-PPO after 1 h (entry **10**, Table 1).

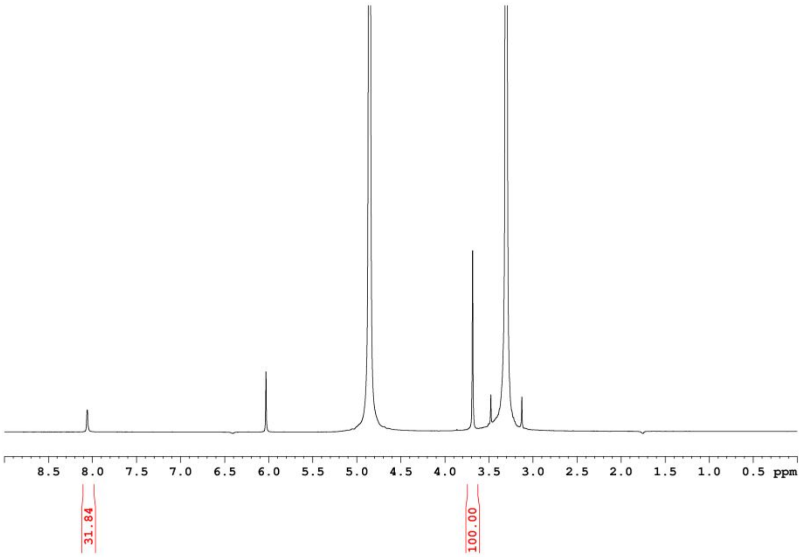

Figure S12. ^{1}H NMR spectrum (400 MHz, methanol-d_4) of a catalytic test of formic acid decomposition promoted by AuNPs-PPO after 24 h (entry **10**, Table 1).

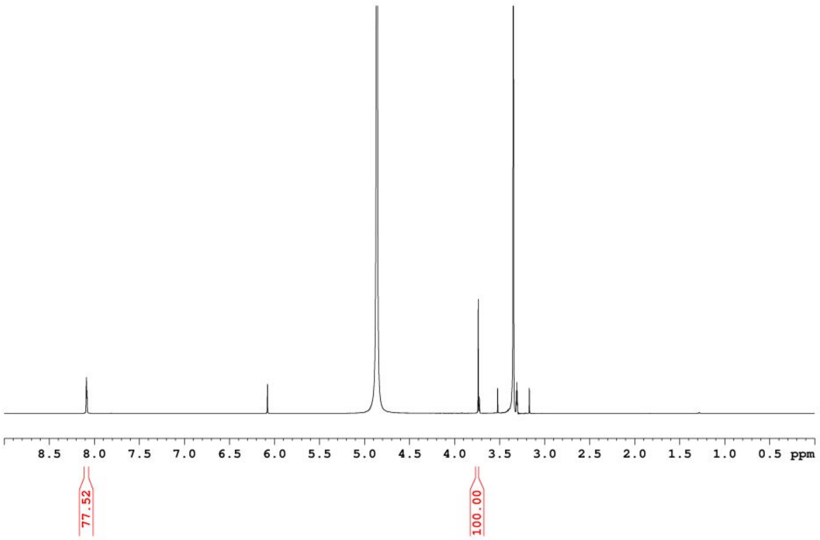

Figure S13. ^{1}H NMR spectrum (400 MHz, methanol-d_4) of a catalytic test of formic acid decomposition promoted by AuNPs-PPO (entry **11**, Table 1).

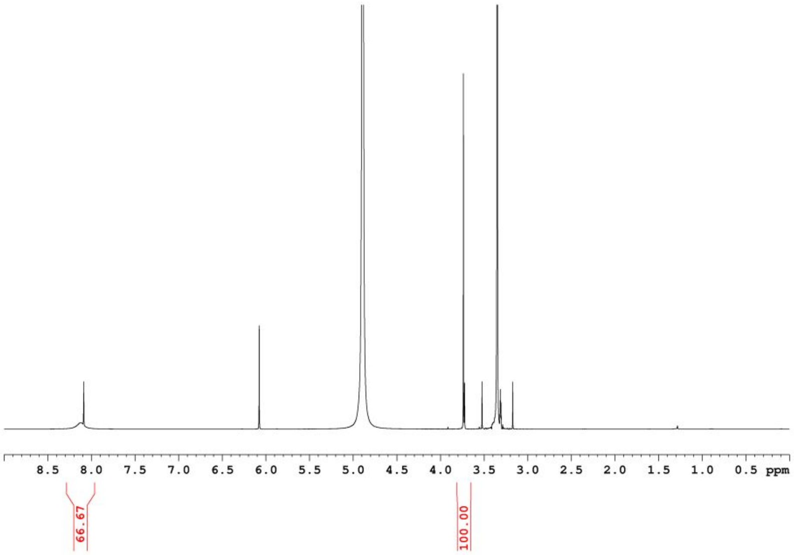

Figure S14. ^{1}H NMR spectrum (400 MHz, methanol-d_4) of a catalytic test of formic acid decomposition promoted by AuNPs-PPO (entry **12**, Table 1).

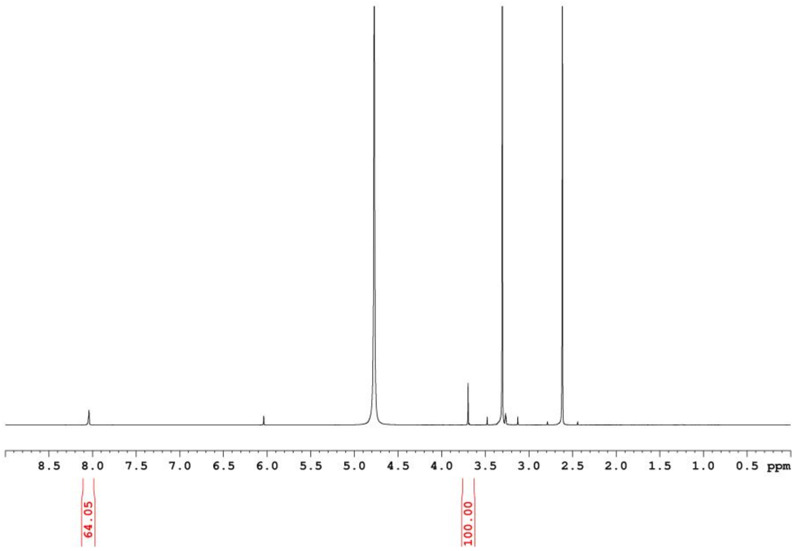

Figure S15. ^{1}H NMR spectrum (400 MHz, methanol-d_4) of a catalytic test of formic acid decomposition promoted by AuNPs-PPO after 24 h (entry **13**, Table 1).

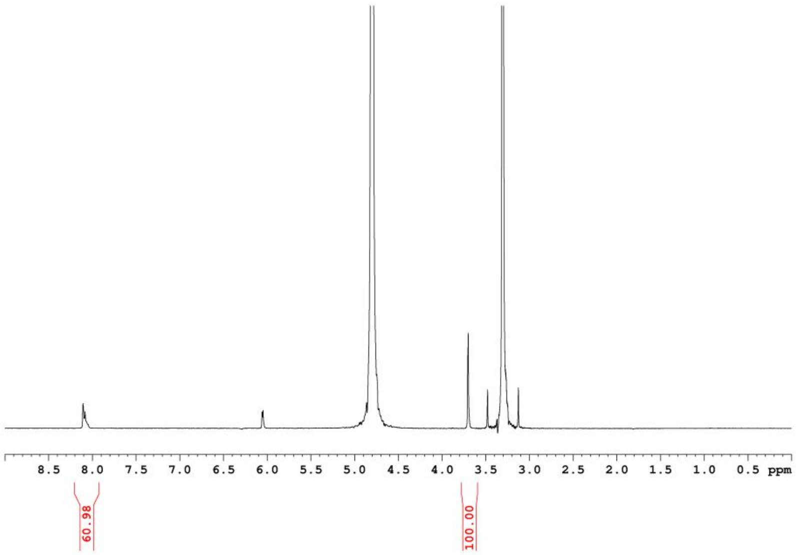

Figure S16. ^{1}H NMR spectrum (400 MHz, methanol-d_4) of a catalytic test of formic acid decomposition promoted by AuNPs-PPO (entry **14**, Table 1).

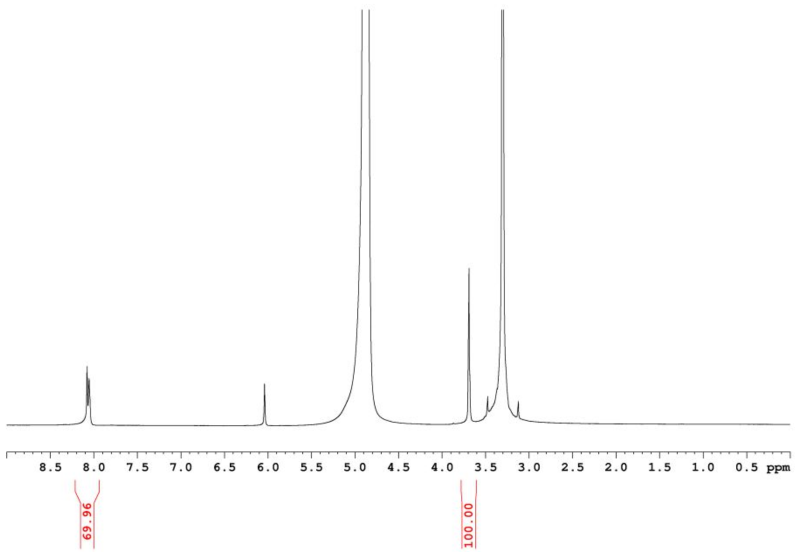

Figure S17. ^{1}H NMR spectrum (400 MHz, methanol-d_4) of a catalytic test of formic acid decomposition promoted by AuNPs-PPO (entry **15**, Table 1).

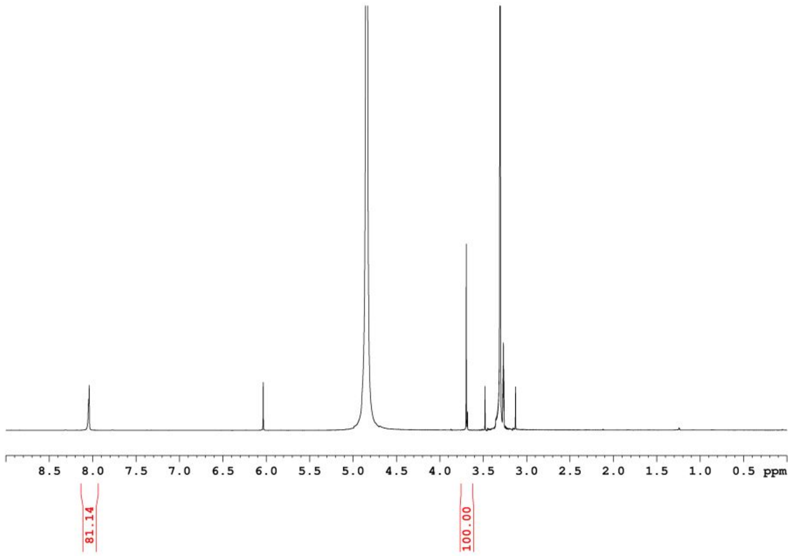

Figure S18. ^{1}H NMR spectrum (400 MHz, methanol-d_4) of a catalytic test of formic acid decomposition promoted by AuNPs-PPO (entry **16**, Table 1).

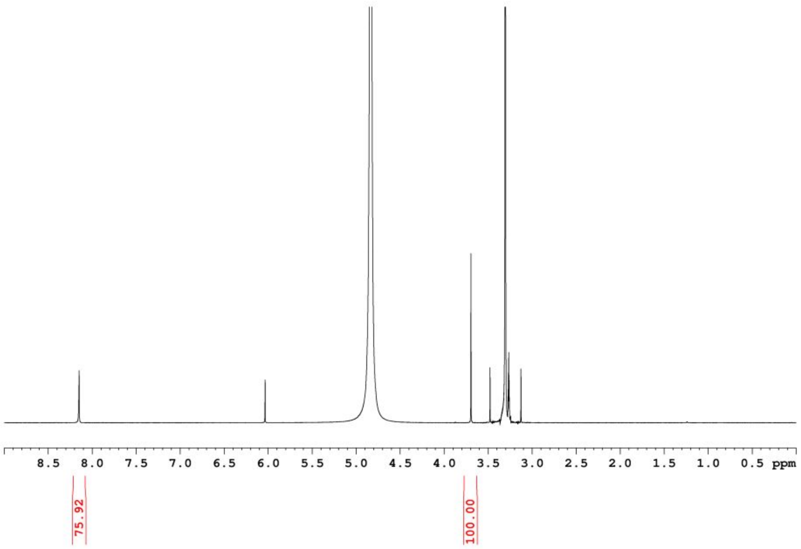

Figure S19. ^{1}H NMR spectrum (400 MHz, methanol-d_4) of a catalytic test of formic acid decomposition promoted by AuNPs-PPO (entry **17**, Table 1).

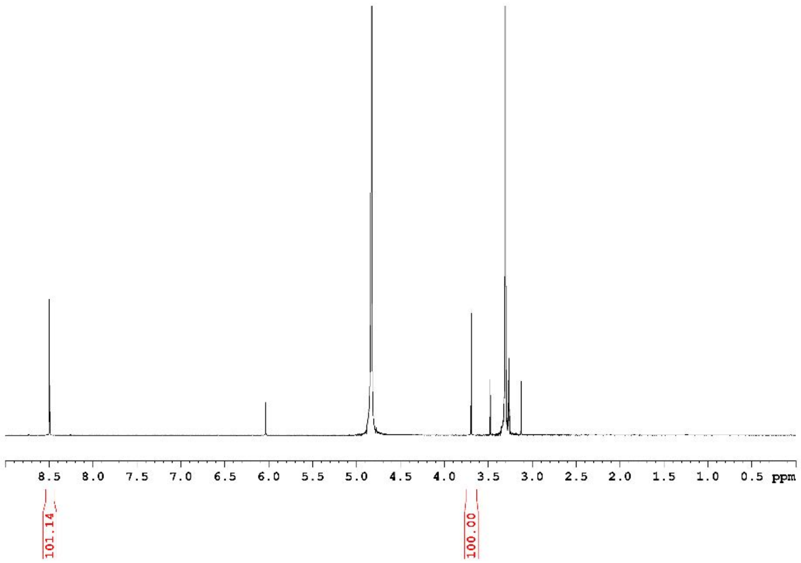

Figure S20. ^{1}H NMR spectrum (400 MHz, methanol-d_4) of a catalytic test of formic acid decomposition promoted by AuNPs-PPO (entry **18**, Table 1).

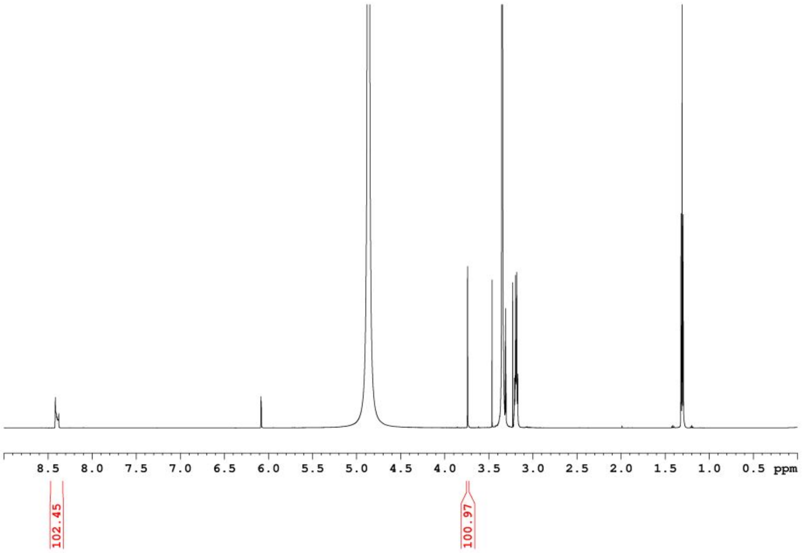

Figure S21. ^{1}H NMR spectrum (400 MHz, methanol-d_4) of a catalytic test of formic acid decomposition promoted by AuNPs-PPO (entry **19**, Table 1).

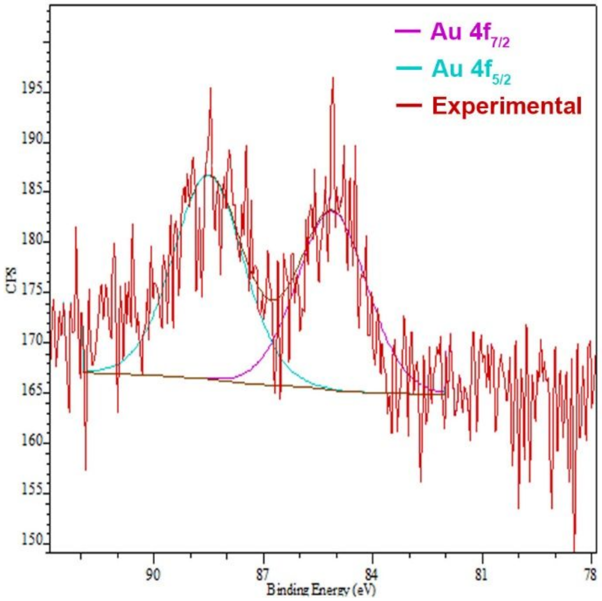

Figure S22. XPS spectrum of AuNPs-PPO catalyst (diagnostic spectral region for gold).
